# Special Issue “Zebrafish: A Model Organism for Human Health and Disease: 2nd Edition”

**DOI:** 10.3390/ijms27209296

**Published:** 2026-10-20

**Authors:** Jyotshna Kanungo

**Affiliations:** Division of Neurotoxicology, National Center for Toxicological Research, U.S. Food and Drug Administration, Jefferson, AR 72079, USA; jyotshnabala.kanungo@fda.hhs.gov

The era of using zebrafish (*Danio rerio*) as a model to investigate human disease began in the 1960s with the pioneering work by a molecular biologist, George Streisinger, who established its role as a genetic vertebrate model [1]. Following his landmark study, the large-scale utility of zebrafish for modeling human disease emerged, leading to extensive developmental genetic screens in the 1990s that detected and matched mutations in zebrafish genes to human disease genes [2,3]. Furthermore, sequencing of the zebrafish genome revealed that roughly 70% to 84% of genes in zebrafish are similar to those in humans, strengthening their utility for modeling specific human diseases.

The transformation of zebrafish from a simple freshwater aquarium pet into one of the most powerful and widely used alternative models in modern biomedical research has been a remarkable scientific journey (Figure 1). The zebrafish model possesses major advantages, such as high fecundity, rapid development, and cost-effective maintenance. A single female lays hundreds of externally fertilized and transparent eggs, increasing the sample size for the experiments, allowing longitudinal assessment of developmental organogenesis and exogenous reporter transgene expressions, and cost-effective experimentation. Zebrafish embryos are a human-relevant model, sharing approximately 84% of genes associated with human diseases. Despite being a lower vertebrate, their major organ systems, developmental pathways, and cellular/molecular mechanisms are highly conserved, and they function similarly to those in humans.

Established as a valuable alternative animal model, zebrafish aligns seamlessly with the three R’s, (reduction, refinement, and replacement) supporting the humane use of animals in biomedical research. Consequently, several compounds identified via zebrafish screens conducted by different laboratories have undergone clinical trials [4]. Current emphasis on alternatives in research involving animals positions the zebrafish model at the heart of new approach methodologies (NAMs). Federal agencies in the USA, including the National Institutes of Health (NIH) and Food and Drug Administration (FDA), consider early stage zebrafish embryos as non-mammalian whole-organism alternatives.

This Special Issue, “Zebrafish: A Model Organism for Human Health and Disease: 2nd Edition”, is a collection of five papers with four original research articles and one comprehensive review. The featured papers underscore the utility of the zebrafish model in recapitulating specific human pathological conditions and elucidating underlying mechanisms that can lead to strategic interventional avenues. The investigators of these papers used both wild-type and genetically modified zebrafish to address tissue-/organ-specific conditions and covered a wide array of topics such as the nervous system, inflammation, and wound healing. Ultimately, these papers leverage the exceptional capabilities of the zebrafish model to provide critical cellular and molecular insights into the specific pathological processes. Such insight can drive forward our understanding of human conditions and potential interventional strategies.

In the review paper, Lemos et al. described the relevance of using rodents as well as alternative animal models, such as fish and nematodes, as experimental models to study inflammation [5]. These models facilitate the efficient investigation of acute and chronic phases of inflammation, and also provide the opportunity, especially with the use of lower phyla organisms, to delineate the mechanisms of inflammation that cannot be studied in humans. Rodents and zebrafish share a higher degree of genomic similarity with humans compared to the nematodes. Nematodes lack adaptive immunity. However, they have been a robust model to study innate immune responses, especially involving host–pathogen interactions. The review article emphasizes the utility of alternate animal models to understand human inflammation underscoring the relief from ethical concerns and financial constraints.

In their paper, Uppalapati et al. described the roles of MMP13 and pro-migratory MMPs (Matrix Metalloproteinases) in collective cell migration (CCM) of zebrafish keratocytes, a process associated with wound healing [6]. MMP2, MMP9, MMP13 and MMP14 are distinct members of the MMPs family, which are enzymes that break down the supportive network of proteins surrounding the cells [7]. The authors used wild-type zebrafish to establish explant cultures for all the assays. The results showed that while MMP13 exhibited anti-migratory effects, MMP2, MMP9 and MMP14 acted as pro-migratory molecules in zebrafish keratocytes. The authors conclude that their study revealed a novel inhibitory role for MMP regulation of CCM, which would have implications in understanding such complex biological processes.

In a study by Lee et al., the authors used a multi-level screening platform in which they screened zebrafish embryos to identify short peptides that improved motor neuron growth [8]. In the *Tg(mnx1:GFP)* transgenic line, green fluorescent protein (GFP) is expressed in the motor neurons and axons that facilitate live monitoring of motor neuron development [9]. The authors synthesized a number of peptides derived from recombinant phosphoglycerate kinase 1 (Pgk1) since supplementation with Pgk1 has been shown to improve motor neuron growth (reviewed in [10]). Based on zebrafish motor neuron phenotype screening, the authors demonstrated that a mutated short peptide (M039) had the most pronounced effect on improving neurite growth among all 17 mutants tested. The therapeutic potential of the short peptides may lead to further studies on the application of these findings to human motor neuron diseases.

In another paper by Gu et al. [11], the authors used zebrafish embryo as an alternative animal model to investigate potential adverse effects of inorganic arsenic in humans. They previously reported that sodium arsenite induced supernumerary motor neuron development and apoptosis in zebrafish embryos and a dietary supplement, and acetyl L-carnitine (ALCAR) abolished apoptosis only but not the abnormal motor neuron development [12,13]. One of the causes of apoptosis is oxidative stress. To delineate the mechanism of arsenic-induced apoptosis and supernumerary motor neuron development, the authors explored the effect of another antioxidant, N-acetylcysteine (NAC). Just like ALCAR, NAC reduced arsenic-induced apoptosis in the zebrafish embryos but did not prevent sodium arsenite-induced supernumerary motor neuron development. These data suggest that arsenic-induced apoptosis and supernumerary motor neuron development are mediated via distinct pathways. They also revealed that protein oxidation (carbonylation) and p53 signaling, a crucial regulator of apoptosis, were not involved in arsenic-induced apoptosis nor supernumerary motor neuron development. The results add to the understanding of arsenic toxicity and diseases in the human population.

Mardhekar et al. [14] investigated the role of intracranial aneurysm-associated COL22A1 (collagen XXII) variants in cerebrovascular structure and barrier integrity in zebrafish. An intracranial aneurysm (IA), a cerebrovascular disorder, is characterized by a weak, balloon-like bulge in intracranial arteries affecting approximately 2–5% of the human population [15]. Despite the knowledge that environmental and genetic factors contribute to IA susceptibility, the genetic basis of IA remains unclear. The authors previously identified several single nucleotide variants of COL22A1 in individuals affected with IA. The current study tested the functional effects of these variants in the zebrafish embryo model. Their study showed that inducible overexpression of each of the six human COL22A1 single nucleotide variants increased the incidence of cranial hemorrhage in the zebrafish embryos, in contrast to overexpression of wild-type COL22A1 that had no significant effect. Overexpression of specific variants of COL22A disrupted intracranial neuro-vasculature integrity. Using in silico modeling, the authors propose a potential mechanism suggesting that the specific variant likely perturbed the triple-helix formation in COL22A, causing protein misfolding, which can lead to its dysfunction. These findings offer a genetic basis of IA that may be applicable to understanding such pathological conditions in humans.

In conclusion, this Special Issue presents a collection of papers that reinforce the successful use of the zebrafish model to investigate human diseases and pathologies. The zebrafish model offers a powerful platform for high-throughput drug discovery and preclinical testing [16,17], thus integrating laboratory research into the translational pipeline. Furthermore, given the rising momentum toward NAMs, which prioritizes tools and technologies that replace or reduce mammalian models in research, zebrafish embryo stands out as a pivotal model for advancing human health/disease research ethically and effectively.

## Figures and Tables

**Figure 1:**
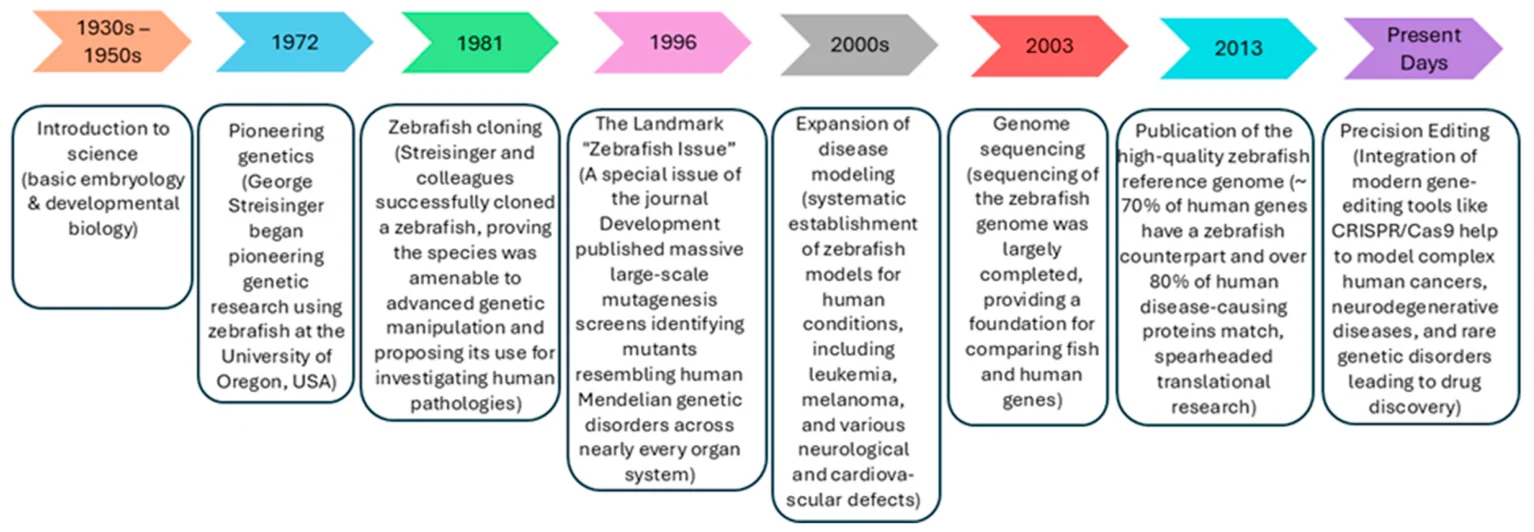
Timeline of discoveries that enabled the specific use of zebrafish as a human disease model.
